# Impact of sepsis with acute kidney injury and acute respiratory distress syndrome on patient prognosis: A multicenter retrospective cohort study

**DOI:** 10.1097/MD.0000000000049743

**Published:** 2026-07-17

**Authors:** Dong-Hui Wang, Jin-Chao Zhao, Xiao-Yun Liu, Xiu-Ming Xi, Feng-Sheng Cao, Wen-Xiong Li

**Affiliations:** a Department of Intensive Critical Unit, Xiangyang No. 1 People’s Hospital, Hubei University of Medicine, Xiangyang, China; b Department of Critical Care Medicine, Fuxing Hospital, Capital Medical University, Beijing, China; c Department of Intensive Critical Unit, Beijing Chao-yang Hospital, Capital Medical University, Beijing, China.

**Keywords:** acute kidney injury, acute respiratory distress syndrome, sepsis

## Abstract

Sepsis, acute kidney injury (AKI), and acute respiratory distress syndrome (ARDS) are severe conditions commonly seen in the intensive care unit (ICU). The study was designed to evaluate the influence of single-organ dysfunction (AKI or ARDS) and 2-organ dysfunction (AKI combined with ARDS) on the 30-day mortality risk in patients with sepsis. Data originated from a prospective multicenter cohort involving 18 Chinese ICUs, which enrolled patients with sepsis following ICU admission. Patients were stratified into 4 groups according to their organ status within 7 days of sepsis onset: those who did not develop AKI or ARDS (non-AKI and non-ARDS group), those who developed AKI but not ARDS (AKI and non-ARDS group), those who developed ARDS but not AKI (ARDS and non-AKI group), and those who developed both AKI and ARDS (AKI and ARDS group). The primary endpoint was defined as mortality within 30 days of sepsis diagnosis. Furthermore, a survival analysis was conducted among the 4 groups. A total of 2175 septic patients were eligible, including 273 cases (12.6%) in the non-AKI and non-ARDS group, 435 cases (20.0%) in the AKI and non-ARDS group, 560 cases (25.7%) in the ARDS and non-AKI group, and 907 cases (41.7%) in the AKI and ARDS group. Kaplan–Meier analysis revealed that the survival probabilities of the 4 groups at 30 days after sepsis diagnosis were 85.6%, 70.6%, 58.9%, and 50.8%, respectively (*P* < .001). Compared with the patients in the non-AKI and non-ARDS group, a landmark analysis showed that the average adjusted hazard ratios for the 30-day mortality risk for the AKI and non-ARDS, ARDS and non-AKI, and AKI and ARDS groups were 2.19 (95% confidence interval [CI]: 1.26–3.82, *P* = .006), 2.92 (95% CI: 1.73–4.93, *P* < .001), and 3.09 (95% CI: 1.85–5.15, *P* < .001), respectively. Septic patients complicated with AKI and ARDS had a poor prognosis, with a 30-day survival rate of only 50.8% after the diagnosis of sepsis.

## 1. Introduction

Defined as a life-threatening organ dysfunction arising from the host’s maladaptive response to infection,^[1]^ sepsis contributes to one-third to one-sixth of deaths worldwide.^[2–4]^ An overactivated immune response during sepsis can lead to cytokine storms, capillary leakage, microcirculation disorders, and ultimately multiple organ dysfunction. Acute kidney injury (AKI) and acute respiratory distress syndrome (ARDS) are common organ dysfunction complications in patients with sepsis.^[5]^ A research report on critically ill patients from 24 European countries reported that the AKI incidence rate among septic patients was 51%, and the intensive care unit (ICU) mortality rate was 41%.^[6]^ According to a recent international multicenter prospective cohort study, up to 10% of patients in ICUs may have ARDS, with mortality rates ranging from 34.9% (mild ARDS) to 46.1% (severe ARDS).^[7]^

The updated Sepsis-3.0 definition places greater emphasis on organ dysfunction and adopts the Sequential Organ Failure Assessment (SOFA) score for sepsis diagnosis. Sepsis complicated by multiple organ dysfunction syndrome (MODS) is associated with high morbidity and mortality.^[8]^ MODS represents the most severe outcome of sepsis.^[9,10]^ The occurrence of organ dysfunction signifies a poor prognosis, serves as a primary cause of death, and contributes to prolonged hospital stays and increased medical costs.^[8]^ The risk of mortality in septic patients increases with both the number of organ failures and the duration of dysfunction. Multiorgan dysfunction is more frequently observed than single-organ failure. A Spanish study reported that 78% of severe sepsis patients in the ICU exhibited multiple organ dysfunctions, while only 22% presented with failure of a single organ.^[10]^ In a multicenter prospective observational study conducted by Kudo et al^[11]^ on the impact of organ dysfunction on mortality in severe sepsis patients, stage 3 AKI emerged as an independent risk factor for elevated mortality, whereas severe ARDS was not. This finding may stem from the modest sample size of the ARDS subgroup (only 37 patients), which might have limited the statistical power to detect significance. In a study of sepsis cases in California from 2008 to 2015, Wardi et al^[12]^ performed multivariate logistic regression analysis on the entire cohort and identified the following factors associated with increased mortality: a greater number of organ failures (odds ratio = 24.28 for ≥3 organs, 8.79 for 2 organs, and 3.32 for 1 organ), age over 65, metastatic cancer, fungal infection, and preexisting liver disease. However, the study did not specify which organ failures were most strongly linked to mortality.

In sepsis-induced multiorgan dysfunction, the lungs are the earliest and most vulnerable target organ.^[13,14]^ However, the incidence and mortality of sepsis with both ARDS and AKI have not yet been reported. Although the incidence and mortality of sepsis patients with AKI or ARDS (or both) are relatively high, the reported incidence rates and mortality rates vary considerably. The impact of septic patients with organ dysfunction (AKI and ARDS) on patient prognosis has not been fully clarified. Thus, we carried out a post hoc analysis of a large, prospective, multicenter cohort study to assess the effect of single-organ dysfunction (AKI or ARDS) and 2-organ dysfunction (AKI combined with ARDS) on the 30-day mortality of septic patients in the ICU. By evaluating the independent and combined impacts of AKI and ARDS on prognosis in a large, multicenter cohort of septic patients, this study aims to provide clinicians with a more nuanced understanding of risk stratification and to inform the design of future interventional trials targeting organ dysfunction in sepsis.

## 2. Materials and methods

### 2.1. Study patients

This study drew on data from a multicenter prospective cohort conducted by the China Critical Care Sepsis Trial (CCCST) working group across 18 Chinese ICUs between January 2014 and August 2015. While the parent CCCST study sought to delineate the epidemiology and clinical features of septic patients, our investigation narrowed its focus to those who, upon ICU admission, fulfilled the Sepsis-3.0 criteria and were thus definitively diagnosed with sepsis.^[1]^ We designated the date of sepsis confirmation as the time of disease onset, after which we prospectively observed the occurrence of AKI and ARDS within the initial 7 days. Exclusion criteria were as follows: preexisting AKI or ARDS on ICU admission; AKI or ARDS that occurred before sepsis; AKI or ARDS that occurred 7 days after the diagnosis of sepsis; chronic kidney disease, prior nephrectomy, or kidney transplantation; renal replacement therapy (RRT) for nonrenal indications; incomplete data records; and age < 18 years.

Written informed consent was obtained from all participants or their legal surrogates. Conducted in compliance with the Declaration of Helsinki, the CCCST protocol received ethical approval (2013FXHEC-KY2018) from the institutional review boards of all participating centers and was registered (ChiCTR-ECH-13003934) with the Chinese Clinical Trial Registry (https://www.chictr.org.cn/).

The patients were categorized into 4 groups within 7 days after a sepsis diagnosis: those who did not develop AKI or ARDS (non-AKI and non-ARDS group), those who developed AKI but not ARDS (AKI and non-ARDS group), those who developed ARDS but not AKI (ARDS and non-AKI group), and those who developed both AKI and ARDS (AKI and ARDS group).

### 2.2. Definitions and study endpoints

The Sepsis-3.0 definition was used to define sepsis.^[1]^ For cases prior to 2016, this definition was applied retrospectively. Septic shock was defined as sepsis that required vasopressors after adequate fluid resuscitation to maintain a mean arterial pressure (MAP) of ≥65 mm Hg and a blood lactate concentration of ≥2 mmol/L.^[1,15]^

The diagnosis and staging of AKI were based on the serum creatinine and urine output criteria proposed by Kidney Disease: Improving Global Outcomes (KDIGO).^[16]^ The definition of baseline serum creatinine was as follows: if at least 5 values were available, the median of all values from 6 months to 6 days before hospitalization was used; otherwise, the minimum value within 5 days before hospitalization was used.^[17]^ If no creatinine value was available before hospitalization or the emergency patient’s serum creatinine was abnormal at admission, baseline creatinine was estimated using the Modification of Diet in Renal Disease equation.^[18]^

ARDS was diagnosed according to the Berlin Definition (2012).^[19]^ At least 2 physicians independently assessed ARDS based on the clinical condition, chest X-ray or computed tomography scans, and arterial blood gases. The severity of ARDS was determined by the arterial partial pressure of oxygen (PaO_2_)/fraction of inspired oxygen (FiO_2_) ratio according to the Berlin classification, using the PaO_2_/FiO_2_ ratio at the time of the first diagnosis of ARDS, namely, mild (200 mm Hg < PaO_2_/FiO_2_ ≤ 300 mm Hg with positive end-expiratory pressure [PEEP] or continuous positive airway pressure ≥ 5 cm H_2_O), moderate (100 mm Hg < PaO_2_/FiO_2_ ≤ 200 mm Hg with PEEP ≥ 5 cm H_2_O), and severe (PaO_2_/FiO_2_ ≤ 100 mm Hg with PEEP ≥ 5 cm H_2_O).

The primary endpoint was 30-day mortality after sepsis diagnosis. The secondary endpoints were the ICU length of stay (LOS), hospital LOS, ICU mortality, and hospital mortality.

### 2.3. Data collection

We extracted patient demographics, including sex, age, body mass index (BMI), baseline comorbidities, admission pathway (medical, surgical, or emergency), and the primary reason for ICU stay, from the medical records. Comorbidities considered included diabetes, hypertension, cancer, chronic liver disease, chronic obstructive pulmonary disease (COPD) or asthma, and cardiovascular disorders. Both the Acute Physiology and Chronic Health Evaluation II (APACHE II) and the SOFA scores were calculated on the day that sepsis was confirmed.

On the day of sepsis diagnosis, we retrieved data on therapeutic interventions, specifically mechanical ventilation and RRT. MAP and septic shock status were assessed daily over the first 7 days after sepsis confirmation. Baseline serum creatinine, along with exposure to nephrotoxic agents (angiotensin-converting enzyme inhibitors, aminoglycosides, nonsteroidal anti-inflammatory drugs, vancomycin, and proton pump inhibitors), was also documented.^[20,21]^ We additionally captured information on the ICU LOS, hospital LOS, ICU mortality, 30-day mortality, and hospital mortality.

### 2.4. Statistical analysis

All statistical analyses were performed using R 4.2.3 (R Project for Statistical Computing). Continuous variables were summarized as medians with interquartile ranges, while categorical variables were expressed as percentages. For intergroup comparisons, the Mann–Whitney *U* test was used for continuous data and the chi-square test for categorical data. Statistical significance was defined as a two-sided *P* < .05.

**Figure 1.**
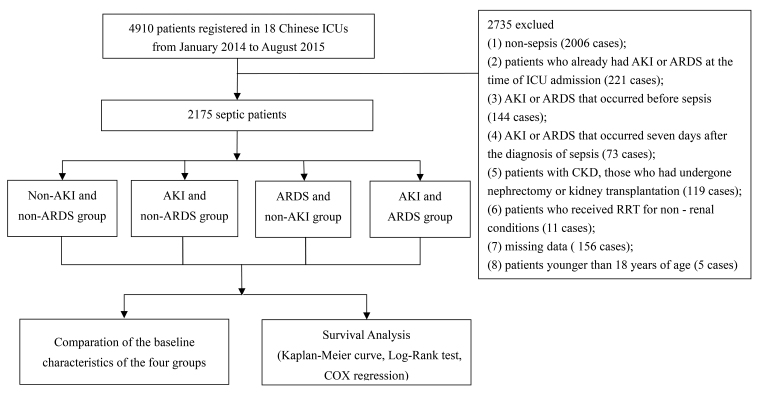
Study flow diagram. AKI = acute kidney injury, ARDS = acute respiratory distress syndrome, CKD = chronic kidney disease, ICU = intensive care unit, RRT = renal replacement therapy.

We performed survival analysis on the 4 groups defined by AKI and ARDS status: the non-AKI and non-ARDS group, the AKI and non-ARDS group, the ARDS and non-AKI group, and the AKI and ARDS group (Fig. 1). The Kaplan–Meier method was employed to assess survival status across the 4 groups, while the log-rank test was applied to evaluate disparities in survival time and rates. The Cox proportional hazards model was additionally employed to derive the hazard ratio (HR) and 95% confidence interval (CI) for 30-day mortality. To avoid overadjustment bias, we optimized covariate selection. The SOFA score was removed due to overlapping components with AKI and ARDS; mechanical ventilation and RRT were excluded as downstream mediators. Adjusting for these would attenuate the total mortality effect. The final model included baseline confounders at sepsis diagnosis: age, sex, BMI, comorbidities, APACHE II score, baseline creatinine, MAP, septic shock, and nephrotoxic drugs. Then, we conducted sensitivity analyses using comprehensive subgroup assessments to verify the robustness of our primary findings across key clinical patient strata.^[22]^ Cox proportional hazards models were systematically reapplied to predefined subgroups, including sex stratification (male vs female), age stratification (<75 vs ≥75 years), and the sequential exclusion of patients receiving RRT or with nephrotoxic drug exposures. These subgroups were selected a priori to assess potential effect modification by demographic factors and to control for confounding related to treatment intensity and pharmacologic exposures. All models preserved the original 4 clinical phenotypes (non-AKI and non-ARDS group, AKI and non-ARDS group, ARDS and non-AKI group, and AKI and ARDS group), using the non-AKI and non-ARDS group as the reference. Consistency of effects was rigorously evaluated based on the direction and magnitude of the HR, as well as the stability of statistical significance across all subgroup analyses.

To address potential immortal time bias, we performed a landmark analysis set at day 7 post-sepsis diagnosis. Patients who died or were censored before day 7 were excluded (censoring included discharge alive, loss to follow-up, or survival to the end of the 30-day study period). Survival time was recalculated from day 7, and multivariable Cox regression was performed using the same covariate adjustment set. Sensitivity analyses were conducted at alternative landmark time points (days 3, 5, and 10) to assess the robustness of our findings. The proportional hazards assumption was tested using Schoenfeld residuals.

## 3. Results

### 3.1. Demographic characteristics and incidence of AKI and ARDS

Among the 4910 screened individuals, 2175 patients with sepsis were ultimately included in our analysis (Table 1). Specifically, 12.6% (273 cases) were in the non-AKI and non-ARDS group, 20.0% (435 cases) were in the AKI and non-ARDS group, 25.7% (560 cases) were in the ARDS and non-AKI group, and 41.7% (907 cases) were in the AKI and ARDS group.

**Table 1:** Baseline characteristics by AKI and/or ARDS status in septic patients.

Variables	Non-AKI and non-ARDS group (n = 273)	AKI and non-ARDS group (n = 435)	ARDS and non-AKI group (n = 560)	AKI and ARDS group (n = 907)	*P*-value
Male, n (%)	176 (64.5%)	244 (56.1%)	362 (64.6%)	606 (66.8%)	.002
Age, median (IQR), yr	63 (48–74)	61 (46–75)	64.5 (50–76)	65 (51–77)	.001
BMI, median (IQR), kg/m^2^	22.5 (20.2–24.5)	22.9 (20.8–25.0)	23.3 (20.8–25.0)	23.4 (21.5–25.6)	<.001
Chronic comorbidities, n (%)					
COPD/asthma	16 (5.9%)	14 (3.2%)	48 (8.6%)	89 (9.8%)	<.001
Cardiovascular disease	42 (15.4%)	69 (15.9%)	66 (11.8%)	143 (15.8%)	.159
Hypertension	75 (27.5%)	138 (31.7%)	163 (29.1%)	311 (34.3%)	.079
Diabetes	48 (17.6%)	81 (18.6%)	99 (17.7%)	169 (18.6%)	.954
Cancer	31 (11.4%)	44 (10.1%)	55 (9.8%)	94 (10.4%)	.922
Chronic liver disease	0 (0%)	7 (1.6%)	11 (2.0%)	22 (2.4%)	.071
Admission type, n (%)					
Medical	143 (52.4%)	254 (58.4%)	298 (53.2%)	518 (57.1%)	<.001
Surgical	86 (31.5%)	110 (25.3%)	115 (20.5%)	159 (17.5%)	<.001
Emergency	44 (16.1%)	71 (16.3%)	147 (26.3%)	230 (25.4%)	<.001
APACHE II score, median (IQR)	16 (11–21)	18 (12–24)	18 (14–24)	21 (15–26)	<.001
SOFA score, median (IQR)	5 (3–7)	7 (4–10)	7 (4–9)	9 (6–12)	<.001
Mechanical ventilation, n (%)	190 (69.6%)	312 (71.7%)	436 (77.9%)	769 (84.8%)	<.001
RRT, n (%)	1 (0.4%)	89 (20.5%)	30 (5.4%)	243 (26.8%)	<.001
Baseline serum creatinine, median (IQR)	78 (60–90)	89 (76–115)	77 (60–94)	91 (72–158)	<.001
Use of nephrotoxic drugs, n (%)	16 (5.9%)	25 (5.8%)	48 (8.6%)	77 (8.5%)	.168
Septic shock, n (%)	93 (34.1%)	232 (53.3%)	219 (39.1%)	523 (57.7%)	<.001
MAP, median (IQR), mm Hg	78 (64–91)	70 (60–83)	79 (66–95)	73 (60–88)	<.001
Outcomes					
LOS in ICU, median (IQR), d	5 (3–13)	7 (4–15)	8 (4–17)	10 (5–18)	.186
LOS in hospital, median (IQR), d	17 (10–28)	17 (9–28)	19 (11–28)	18 (10–28)	<.001
ICU mortality, n (%)	19 (7.0%)	59 (13.6%)	59 (10.5%)	200 (22.1%)	<.001
30-d mortality, n (%)	28 (10.3%)	88 (20.2%)	145 (25.9%)	329 (36.3%)	<.001
Hospital mortality, n (%)	45 (16.5%)	116 (26.7%)	199 (35.5%)	428 (47.2%)	<.001

AKI = acute kidney injury, APACHE II = Acute Physiology and Chronic Health Evaluation II, ARDS = acute respiratory distress syndrome, BMI = body mass index, COPD = chronic obstructive pulmonary disease, ICU = intensive care unit, IQR = interquartile range, LOS = length of stay, MAP = mean arterial pressure, RRT = renal replacement therapy, SOFA = sequential organ failure assessment.

The AKI and ARDS group had a higher age and BMI (*P* < .001). In terms of chronic comorbidities, the AKI and ARDS group had a higher proportion of COPD or asthma, at 9.8% (*P* < .001). Among the chronic comorbidities, there was no significant difference among the 4 groups in cardiovascular disease, hypertension, diabetes, cancer, and chronic liver disease (*P* > .05). Among the admission categories, the proportion of patients admitted from internal medicine was relatively high, at 52.4%, 58.4%, 53.2%, and 57.1%, respectively. The APACHE II and SOFA scores of the AKI and ARDS group were higher (*P* < .001), with scores of 21 (15–26) and 9 (6–12), respectively. The proportion of patients requiring mechanical ventilation, RRT, and septic shock was also relatively high (*P* < .001), at 84.8%, 26.8%, and 57.7%, respectively. There was no statistically significant difference in the length of ICU stay among the 4 groups of patients (*P* > .05). The ARDS and non-AKI group had a higher 30-day mortality rate and hospital mortality rate than the AKI and non-ARDS group. The ICU mortality rate, 30-day mortality rate, and hospital mortality rate were the highest in the AKI and ARDS group (*P* < .001), with rates of 22.1%, 36.3%, and 47.2%, respectively.

### 3.2. Characteristics classified by 30-day survival status in septic patients

In Table 2, there was no statistically significant difference (*P* > .05) between the survival group and the death group in terms of sex, BMI, and a history of chronic liver disease. COPD/asthma, cardiovascular disease, hypertension, diabetes, and cancer were significantly more prevalent among non-survivors than among survivors. The APACHE II and SOFA scores of the death group were higher than those of the sepsis survival group, with scores of 25 (19–29) and 9 (6–12), respectively. Mechanical ventilation, RRT, and septic shock were more frequently observed in non-survivors than in survivors. In the death group, the proportion of patients with both AKI and ARDS was significantly higher than in the survival group, at 55.8% (*P* < .001). AKI staging and ARDS severity are presented for cohort description only.

**Table 2:** Baseline characteristics classified by 30-day survival status in septic patients.

Variables	Survival group (n = 1585)	Non-survival group (n = 590)	*P*-value
Male, n (%)	1006 (63.5%)	382 (64.7%)	.582
Age, median (IQR), yr	61 (46–72)	75 (63–84)	<.001
BMI, median (IQR), kg/m^2^	23.3 (20.9–25.2)	23.3 (20.9–25.4)	.912
Chronic comorbidities, n (%)			
COPD/asthma	101 (6.4%)	66 (11.2%)	<.001
Cardiovascular disease	194 (12.2%)	126 (21.4%)	<.001
Hypertension	443 (27.9%)	244 (41.4%)	<.001
Diabetes	257 (16.2%)	140 (23.7%)	<.001
Cancer	150 (9.5%)	74 (12.5%)	.036
Chronic liver disease	26 (1.6%)	14 (2.4%)	.258
Admission type, n (%)			
Medical	857 (54.1%)	356 (60.3%)	<.001
Surgical	409 (25.8%)	61 (10.3%)	
Emergency	319 (20.1%)	173 (29.3%)	
APACHE II score, median (IQR)	17 (12–22)	25 (19–29)	<.001
SOFA score, median (IQR)	7 (4–10)	9 (6–12)	<.001
Mechanical ventilation, n (%)	1206 (76.1%)	501 (84.9%)	<.001
RRT, n (%)	192 (12.1%)	171 (29.0%)	<.001
Baseline serum creatinine, median (IQR)	84 (68–106)	91 (72–159)	<.001
Use of nephrotoxic drugs, n (%)	92 (5.8%)	74 (12.5%)	<.001
Septic shock, n (%)	704 (44.4%)	363 (61.5%)	<.001
MAP, median (IQR), mm Hg	76 (62–90)	70 (59–87)	<.001
ARDS, n (%)			
Mild ARDS	416 (26.2%)	147 (24.9%)	<.001
Moderate ARDS	445 (28.1%)	219 (37.1%)	
Severe ARDS	132 (8.3%)	108 (18.3%)	
AKI, n (%)			
Stage 1 AKI	365 (23.0%)	109 (18.5%)	<.001
Stage 2 AKI	290 (18.3%)	111 (18.8%)	
Stage 3 AKI	270 (17.0%)	197 (33.4%)	
ARDS and AKI	578 (36.4%)	329 (55.8%)	<.001

AKI = acute kidney injury, APACHE II = Acute Physiology and Chronic Health Evaluation II, ARDS = acute respiratory distress syndrome, BMI = body mass index, COPD = chronic obstructive pulmonary disease, IQR = interquartile range, MAP = mean arterial pressure, RRT = renal replacement therapy, SOFA = sequential organ failure assessment.

### 3.3. Survival analysis

**Figure 2.**
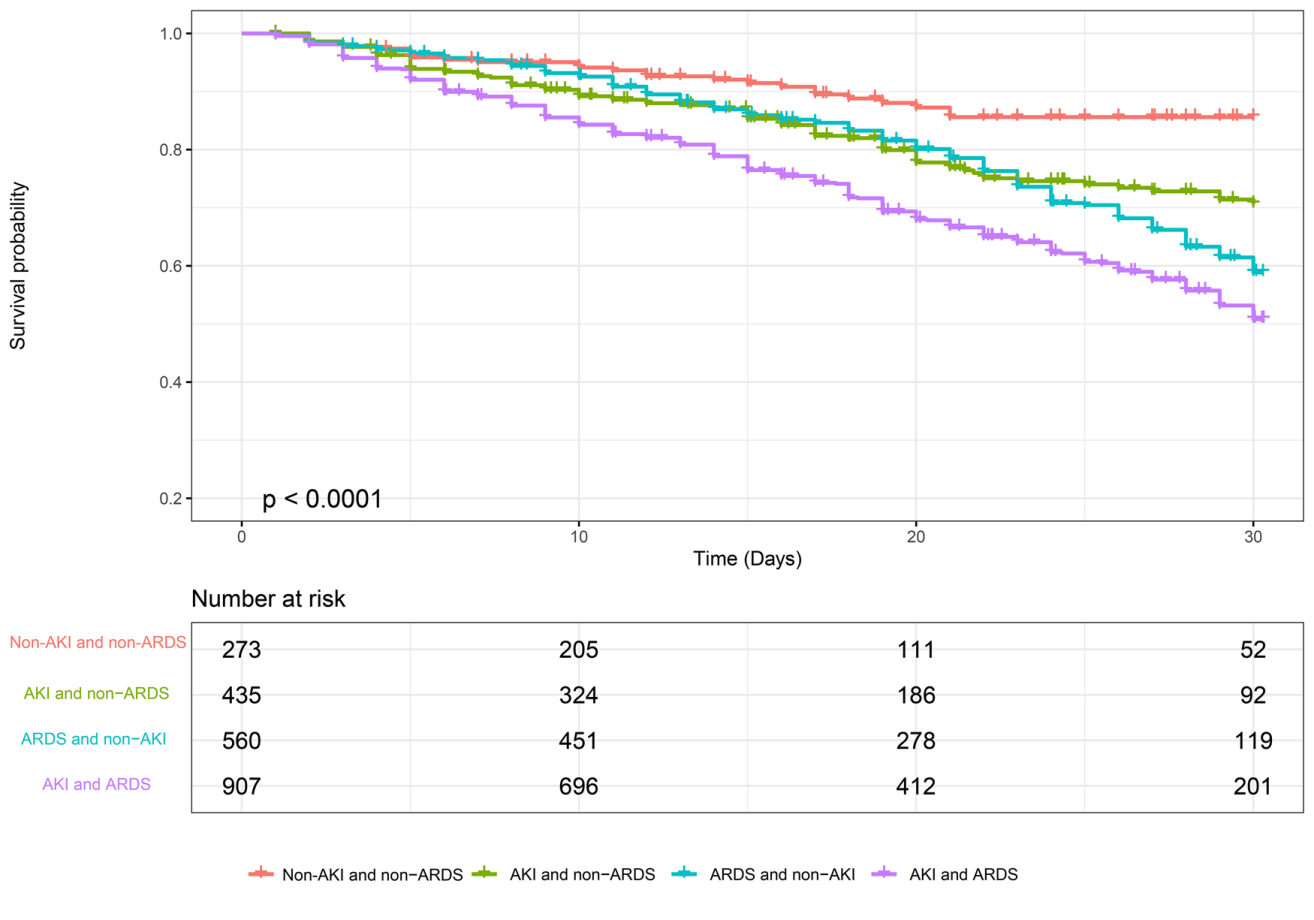
Kaplan–Meier survival analysis. AKI = acute kidney injury, ARDS = acute respiratory distress syndrome.

**Figure 3.**
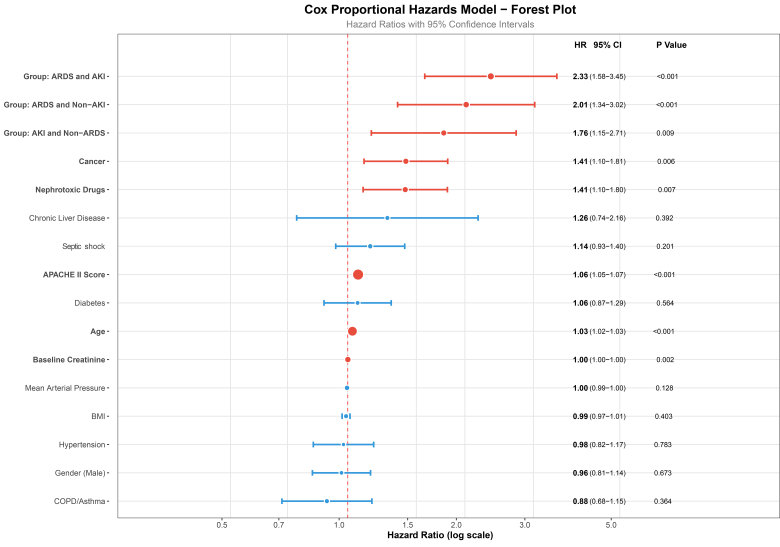
Forest plot of multivariable Cox proportional hazards regression analysis for 30-day mortality stratified by AKI and ARDS status in septic patients. AKI = acute kidney injury, APACHE II = Acute Physiology and Chronic Health Evaluation II, ARDS = acute respiratory distress syndrome, BMI = body mass index, CI = confidence interval, COPD = chronic obstructive pulmonary disease, HR = hazard ratio.

In Figure 2, Kaplan–Meier analysis showed that the survival probabilities of the 4 groups at 30 days after the diagnosis of sepsis were 85.6% (95% CI: 80.6%–91.0%), 70.6% (95% CI: 65.2%–76.5%), 58.9% (95% CI: 53.5%–64.8%), and 50.8% (95% CI: 46.8%–55.2%), respectively (*P* < .001). Compared with the patients in the non-AKI and non-ARDS group, multivariate Cox regression showed that the adjusted HRs for the 30-day mortality risk were 1.76 (95% CI: 1.15–2.71, *P* = .009), 2.01 (95% CI: 1.34–3.02, *P* < .001), and 2.33 (95% CI: 1.58–3.45, *P* < .001), respectively (Fig. 3). Supplemental Material Table S1, Supplemental Digital Content 2, shows the multivariable Cox proportional hazards regression analysis for 30-day mortality stratified by AKI and/or ARDS status in septic patients.

### 3.4. Sensitivity analysis

**Figure 4.**
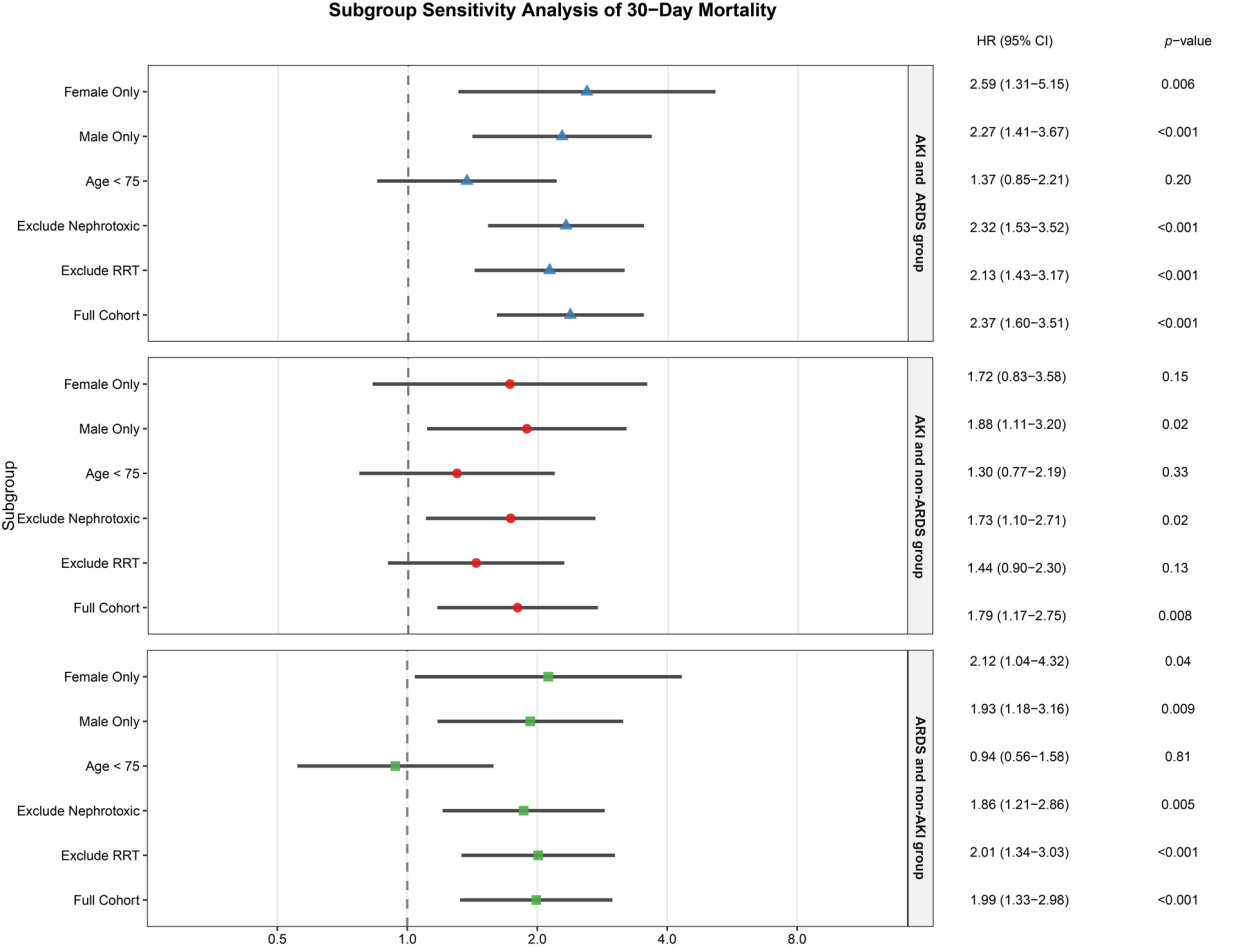
Forest plot of subgroup sensitivity analysis of 30-day mortality. AKI = acute kidney injury, ARDS = acute respiratory distress syndrome, CI = confidence interval, HR = hazard ratio, RRT = renal replacement therapy.

In Figure 4, subgroup analyses confirmed the robustness of the associations between clinical phenotypes and 30-day mortality. The AKI and ARDS group consistently demonstrated the highest mortality risk across all analytical frameworks, with statistically significant HRs in the full cohort (HR = 2.37, 95% CI: 1.60–3.51, *P* < .001) that remained significant after excluding patients receiving RRT (HR = 2.13, 95% CI: 1.43–3.17, *P* < .001) and those exposed to nephrotoxic drugs (HR = 2.32, 95% CI: 1.53–3.52, *P* < .001).

The ARDS and non-AKI group maintained a significantly elevated risk across subgroups (full cohort: HR = 1.99, 95% CI: 1.33–2.98, *P* = .001; without RRT: HR = 2.01, 95% CI: 1.34–3.03, *P* < .001). In contrast, the AKI and non-ARDS group showed variable associations, with attenuated effects after excluding RRT recipients (HR = 1.44, 95% CI: 0.90–2.30, *P* = .131) and in patients younger than 75 years (HR = 1.30, 95% CI: 0.77–2.19, *P* = .327). The non-AKI and non-ARDS group served as the reference category in all analyses.

### 3.5. Landmark analysis

**Figure 5.**
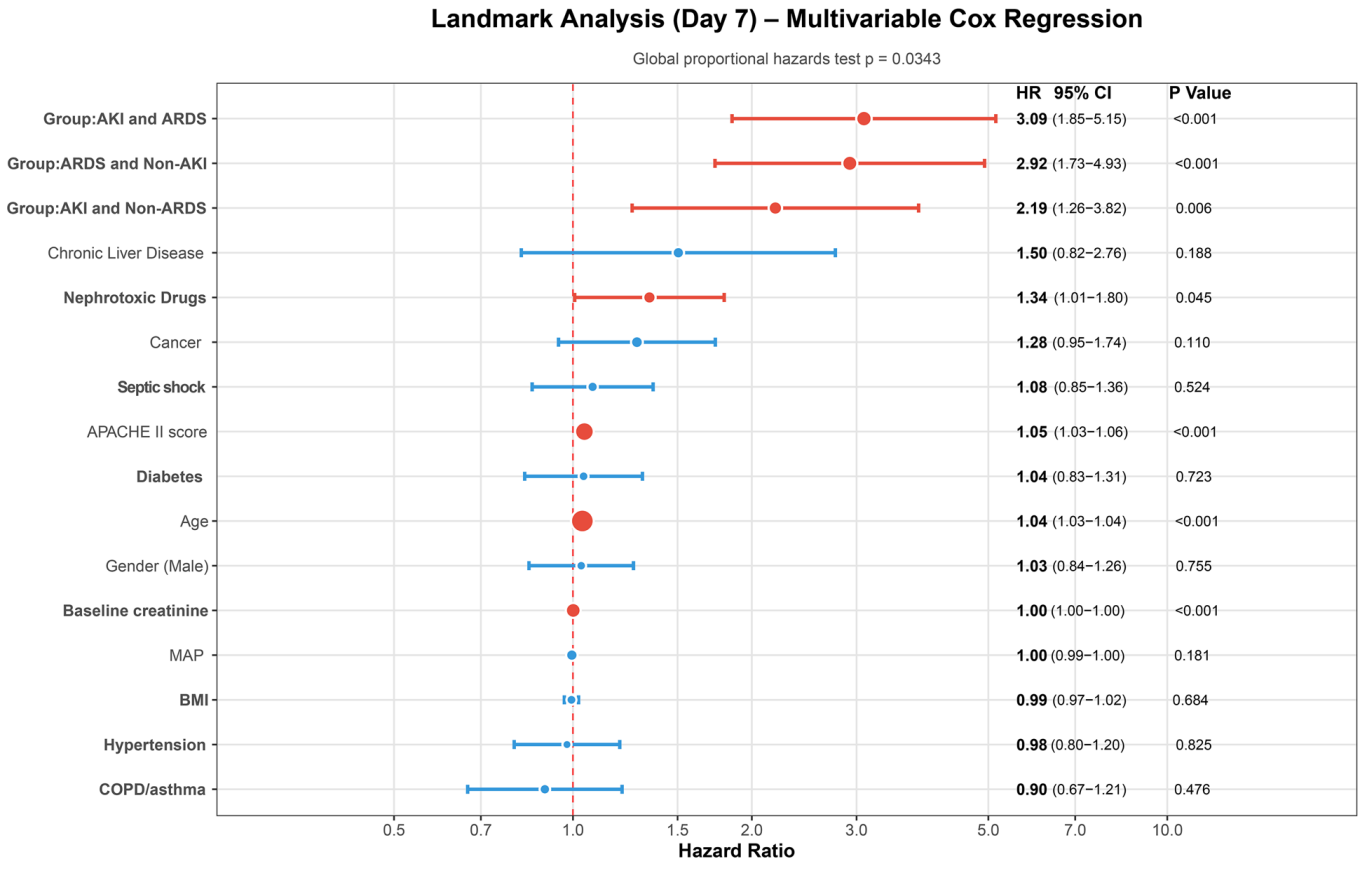
Landmark analysis forest plot. AKI = acute kidney injury, APACHE II = Acute Physiology and Chronic Health Evaluation II, ARDS = acute respiratory distress syndrome, BMI = body mass index, CI = confidence interval, COPD = chronic obstructive pulmonary disease, HR = hazard ratio, MAP = mean arterial pressure.

In the landmark analysis (Fig. 5), we excluded 304 patients (14.0%) who had a follow-up duration of <7 days; of these, 152 died within the first week, while the remaining 152 were censored. Ultimately, 1871 patients were included. The database did not capture the precise date of discharge, precluding the determination of how many censored patients were discharged alive before day 7. The 30-day mortality rates from day 7 were 6.8% in the non-AKI and non-ARDS group, 16.1% in the AKI and non-ARDS group, 24.6% in the ARDS and non-AKI group, and 31.2% in the AKI and ARDS group. After multivariable adjustment, the average HRs for 30-day mortality were 2.19 (95% CI: 1.26–3.82, *P* = .006), 2.92 (95% CI: 1.73–4.93, *P* < .001), and 3.09 (95% CI: 1.85–5.15, *P* < .001), respectively. Sensitivity analyses at different landmark time points yielded consistent results (Supplemental Material Table S2, Supplemental Digital Content 3, and Supplemental Material Fig. 1, Supplemental Digital Content 1).

### 3.6. Schoenfeld residuals test

Schoenfeld residual tests indicated a violation of the proportional hazards assumption in the full-cohort Cox model (global *P* < .001), a finding that is not unexpected in a heterogeneous population of critically ill patients with sepsis. The landmark Cox model (starting at day 7) also violated the assumption (global *P* = .034). Despite this, the landmark analysis is presented as the primary analysis because it circumvents immortal time bias and, crucially, its primary inference is based on the log-rank test (*P* < .001), which does not require the proportional hazards assumption (Supplemental Material Table S3, Supplemental Digital Content 4).

## 4. Discussion

In this multicenter prospective cohort study of 2175 septic patients, we found that combined AKI and ARDS were associated with significantly higher 30-day mortality than either condition alone. The stepwise increase in mortality – from AKI alone to ARDS alone to both – suggests a cumulative burden of organ dysfunction that exceeds additive effects. After multivariable adjustment, patients with combined organ failure had a more than 2-fold increased risk of death, and this association remained robust in sensitivity analyses, including the landmark analysis at day 7 (HR = 3.09, 95% CI: 1.85–5.15).

Further evidence indicates that the failure of specific organs, such as the lungs and kidneys, is associated with particularly high mortality.^[23,24]^ Our study demonstrates that septic patients with ARDS have a worse prognosis than those with AKI alone (*P* < .001). Moreover, those with both AKI and ARDS exhibit higher mortality and poorer outcomes compared with patients with either condition alone (*P* < .001). Our multivariate Cox regression findings align with those of Kudo et al,^[11]^ who reported an AKI incidence of 41.7% (with 31.8% mortality) and an ARDS incidence of 18.7% (with 36.4% mortality) among patients with severe sepsis.

Sepsis can simultaneously trigger both AKI and ARDS. Nevertheless, there is an interaction between AKI and ARDS.^[25,26]^ The possible mechanisms by which ARDS induces AKI include mechanical ventilation, hypoxemia, and systemic inflammation.^[27]^ Meanwhile, the mechanism through which AKI leads to ARDS may be associated with immune-mediated effects, fluid retention, and electrolyte abnormalities.^[28]^ The remarkably consistent association between combined AKI and ARDS and increased mortality necessitates early recognition and timely intervention for ARDS or AKI. For ARDS, a low tidal volume ventilation strategy combined with a conservative fluid management strategy may help prevent the development of AKI in patients with ARDS.^[29]^ However, for patients with AKI, optimizing fluid management, controlling the inflammatory response, appropriately using RRT, and adopting lung-protective ventilation strategies could help prevent ARDS.^[30]^

Clinically, our findings have important implications. First, the markedly worse prognosis of patients with both AKI and ARDS underscores the need for heightened vigilance in this subgroup. Early identification and integrated management of MODS should be prioritized. Second, our results highlight that AKI and ARDS should not be viewed as isolated events but as interconnected syndromes. Future interventional trials should specifically target this high-risk population rather than excluding patients with multiorgan failure, as is often done. Third, our findings reinforce the concept that interventions that preserve function in one organ may have distant beneficial effects, suggesting that strategies targeting shared pathways warrant further investigation.^[25]^

This study had the following strengths. First, reliable data were obtained from a large, multicenter, prospective clinical study conducted in China. Second, we used the Sepsis-3.0 definition and the Berlin criteria for ARDS. However, there were also a few limitations. First, the lack of daily AKI/ARDS onset and discharge dates prevented time-dependent Cox regression and exact identification of 152 censored patients. However, a day-7 landmark analysis avoided immortal time bias. The proportional hazards assumption was violated, but the log-rank test and average HRs provide valid inference. Thus, our conclusions remain robust. Second, while we carefully selected covariates to avoid overadjustment by excluding the SOFA score and potential mediators, residual confounding from unmeasured variables cannot be completely excluded. Third, center-specific identifiers were unavailable in our analytical dataset, precluding adjustment for potential clustering effects across the 18 participating ICUs. However, all centers followed standardized sepsis management protocols based on the Surviving Sepsis Campaign guidelines, which may have reduced inter-center practice variation. Fourth, the use of Modification of Diet in Renal Disease-estimated baseline creatinine may introduce non-differential misclassification, potentially biasing results toward the null and making our findings conservative. Fifth, AKI staging and ARDS severity were presented for descriptive purposes only, as the primary aim was to evaluate the impact of the presence rather than the severity of organ dysfunction. Dose-response relationships and competing risks (e.g., discharge alive before 30 days) warrant investigation in future studies. Finally, while our multicenter design enhances generalizability, all ICUs were from China, which may limit applicability to other healthcare settings.

## 5. Conclusion

The multicenter study demonstrates that septic patients with combined AKI and ARDS have a substantially higher risk of 30-day mortality than those with single-organ dysfunction. These findings highlight the need for integrated organ support strategies and targeted clinical trials in this high-risk population. Future research should focus on elucidating the mechanisms underlying organ crosstalk and evaluating interventions specifically designed for patients with multiorgan failure.

## Acknowledgments

We greatly appreciated the support and assistance offered by all the research centers in the CCCST working group. These 18 ICUs in China included the Department of Critical Care Medicine, Fuxing Hospital, Capital Medical University, Beijing, China; the Department of Critical Care Medicine, West China Hospital, Sichuan University, Sichuan, China; the Medical Intensive Care Unit, Peking Union Medical College Hospital, Beijing, China; the Department of Critical Care Medicine, Guangdong Geriatric Institute, Guangdong General Hospital, Guangdong, China; the Department of Critical Care Medicine, The First Affiliated Hospital of China Medical University, Shenyang, China; the Surgical Intensive Care Unit, Department of Anaesthesiology, Zhongshan Hospital, Fudan University, Shanghai, China; the Intensive Care Unit, The First Hospital of Jilin University, Changchun, China; the Department of Critical Care Medicine, China-Japan Friendship Hospital, Beijing, China; the Department of Critical Care Medicine, Beijing Friendship Hospital, Capital Medical University, Beijing, China; the Surgical Intensive Care Unit, Beijing Chaoyang Hospital, Capital Medical University, Beijing, China; the Department of Respiratory and Critical Care Medicine, Beijing Institute of Respiratory Medicine, Beijing Chaoyang Hospital, Capital Medical University, Beijing, China; the Department of Critical Care Medicine, General Hospital of Ningxia Medical University, Ningxia, China; the Department of Critical Care Medicine, Xiangya Hospital, Central South University, Changsha, China; the Department of Critical Care Medicine, Beijing Tsinghua Changgung Hospital, Beijing, China; the Department of Critical Care Medicine, Beijing Tongren Hospital, Capital Medical University, Beijing, China; the Department of Critical Care Medicine, Peking University Third Hospital, Beijing, China; the Surgical Intensive Care Unit, Xuanwu Hospital, Capital Medical University, Beijing, China; and the Department of Critical Care Medicine, Beijing Tiantan Hospital, Capital Medical University, Beijing, China.

## Author contributions

**Methodology:** Feng-Sheng Cao.

**Formal analysis:** Xiu-Ming Xi.

**Resources:** Xiu-Ming Xi.

**Project administration:** Feng-Sheng Cao, Wen-Xiong Li.

**Validation:** Xiao-Yun Liu.

**Writing – original draft:** Dong-Hui Wang.

**Writing – review & editing:** Jin-Chao Zhao.








