# Supplementary figures and images for: Impact of sepsis with acute kidney injury and acute respiratory distress syndrome on patient prognosis: A multicenter retrospective cohort study
Source: Medicine (Baltimore). 2026 Jul 17;105(29):e49743. doi: 10.1097/MD.0000000000049743 (PMC13384718; doi:10.1097/MD.0000000000049743)

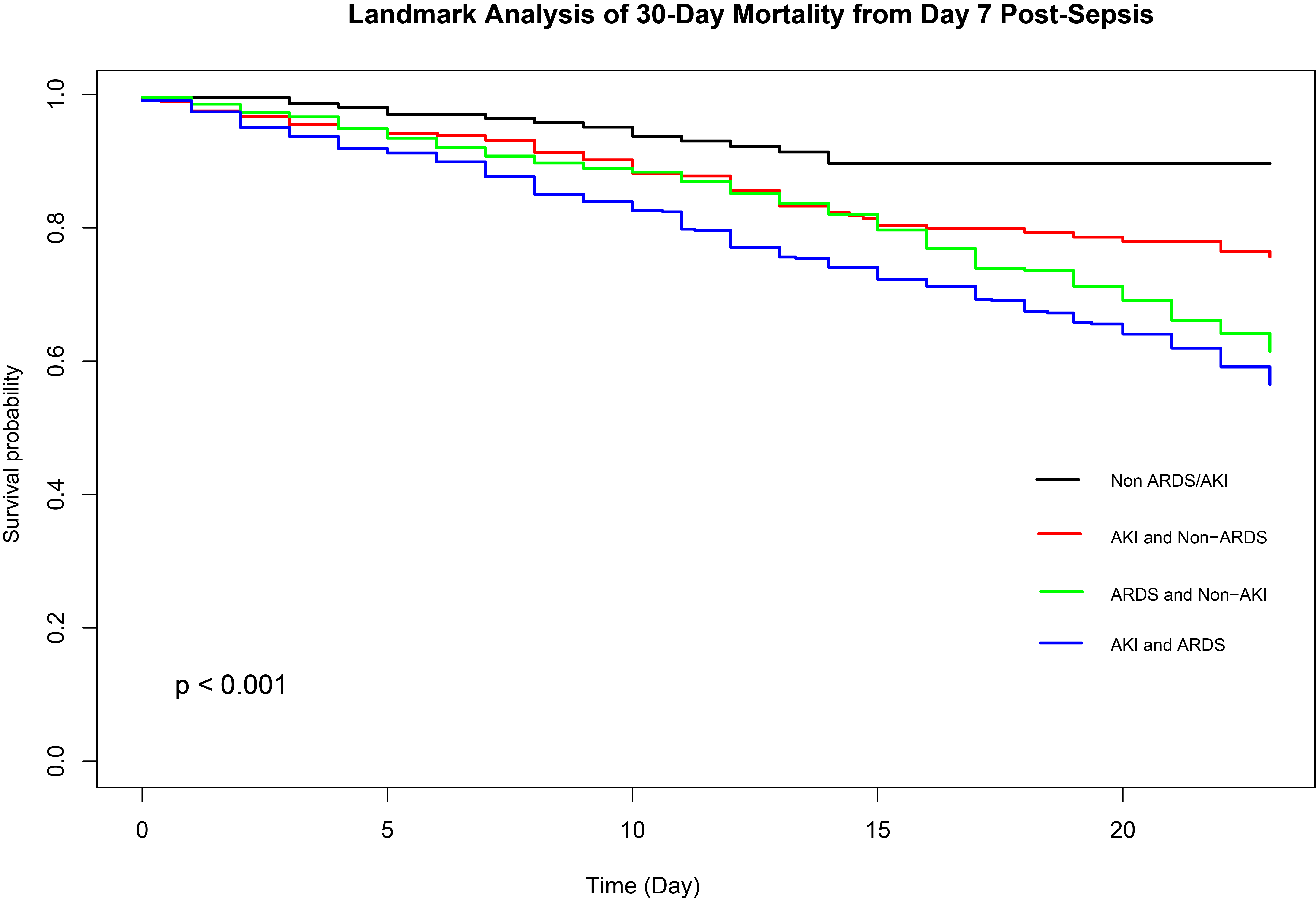

Supplement: Supplementary file 1 [file medi-105-e49743-s001.tif]
