# Supplementary material for: Impact of sepsis with acute kidney injury and acute respiratory distress syndrome on patient prognosis: A multicenter retrospective cohort study
Source: Medicine (Baltimore). 2026 Jul 17;105(29):e49743. doi: 10.1097/MD.0000000000049743 (PMC13384718; doi:10.1097/MD.0000000000049743)
Supplement: Supplementary file 2 [file medi-105-e49743-s002.doc]

**Supplemental Material Table S1. The multivariable Cox proportional hazard regression analysis for 30-day mortality stratified by AKI and/or ARDS status in septic patients**

| **Variables** | **HR** | **95% CI** | ***p-*value** |
| --- | --- | --- | --- |
| Groups | | | |
| Non-AKI and non-ARDS group | 1.00 | - | - |
| AKI and non-ARDS group | 1.76 | 1.15 ~ 2.71 | 0.009 |
| ARDS and non-AKI group | 2.01 | 1.34 ~ 3.02 | < 0.001 |
| AKI and ARDS group | 2.33 | 1.58 ~ 3.45 | < 0.001 |
| Male | 0.96 | 0.81 ~ 1.14 | 0.673 |
| Age | 1.03 | 1.02 ~ 1.03 | < 0.001 |
| BMI | 0.99 | 0.97 ~ 1.01 | 0.403 |
| COPD/asthma | 0.88 | 0.68 ~ 1.15 | 0.364 |
| Hypertension | 0.98 | 0.82 ~ 1.17 | 0.783 |
| Diabetes | 1.06 | 0.87 ~ 1.29 | 0.564 |
| Cancer | 1.41 | 1.10 ~ 1.80 | 0.006 |
| Chronic liver disease | 1.26 | 0.74 ~ 2.16 | 0.392 |
| Septic shock | 1.14 | 0.93 ~ 1.40 | 0.201 |
| Use of nephrotoxic drugs | 1.41 | 1.10 ~ 1.80 | 0.007 |
| Baseline serum creatinine | 1.00 | 1.00 ~ 1.00 | 0.002 |
| MAP | 1.00 | 0.99 ~ 1.00 | 0.128 |
| APACHE II score | 1.06 | 1.05 ~ 1.07 | < 0.001 |

Abbreviations: AKI, acute kidney injury; ARDS, acute respiratory distress syndrome; BMI, body mass index; COPD, chronic obstructive pulmonary disease; MAP, mean arterial pressure; APACHE II, acute physiologic and chronic health evaluation II
