# Supplementary material for: Impact of sepsis with acute kidney injury and acute respiratory distress syndrome on patient prognosis: A multicenter retrospective cohort study
Source: Medicine (Baltimore). 2026 Jul 17;105(29):e49743. doi: 10.1097/MD.0000000000049743 (PMC13384718; doi:10.1097/MD.0000000000049743)
Supplement: Supplementary file 3 [file medi-105-e49743-s003.doc]

**Supplemental Material Table S2**. **Sensitivity Analysis at Different Landmark Time Points**

Abbreviations: AKI, acute kidney injury; ARDS, acute respiratory distress syndrome

| **Landmark Time** | **AKI and non-ARDS group HR (95% CI)** | ***p***-value | **ARDS and non-AKI group HR (95% CI)** | ***p***-value | **AKI and ARDS group HR (95% CI)** | ***p***-value |
| --- | --- | --- | --- | --- | --- | --- |
| Day 3 | 1.95 (1.23–3.08) | 0.004 | 2.20 (1.43–3.40) | <0.001 | 2.65 (1.74–4.04) | <0.001 |
| Day 5 | 1.97 (1.21–3.21) | 0.007 | 2.36 (1.49–3.74) | <0.001 | 2.65 (1.69–4.16) | <0.001 |
| Day 7 | 2.19 (1.26–3.82) | 0.006 | 2.92 (1.73–4.93) | <0.001 | 3.09 (1.85–5.15) | <0.001 |
| Day 10 | 1.92 (1.07–3.44) | 0.029 | 2.81 (1.64–4.83) | <0.001 | 2.93 (1.72–4.97) | <0.001 |
