# Supplementary material for: Impact of sepsis with acute kidney injury and acute respiratory distress syndrome on patient prognosis: A multicenter retrospective cohort study
Source: Medicine (Baltimore). 2026 Jul 17;105(29):e49743. doi: 10.1097/MD.0000000000049743 (PMC13384718; doi:10.1097/MD.0000000000049743)
Supplement: Supplementary file 4 [file medi-105-e49743-s004.doc]

**Supplemental Material Table S3 Schoenfeld Residuals Test for proportional hazards assumption in the full‑cohort and day‑7 landmark Cox models**

| **Model** | **Variable** | **χ²** | **df** | **p-value** |
| --- | --- | --- | --- | --- |
| Full cohort | group | 20.70 | 3 | <0.001 |
| Full cohort | gender | 2.27 | 1 | 0.132 |
| Full cohort | age | 15.60 | 1 | <0.001 |
| Full cohort | BMI | 2.10 | 1 | 0.147 |
| Full cohort | COPD/asthma | 0.01 | 1 | 0.941 |
| Full cohort | hypertension | 0.19 | 1 | 0.666 |
| Full cohort | diabetes | 1.81 | 1 | 0.179 |
| Full cohort | cancer | 1.76 | 1 | 0.185 |
| Full cohort | chronic liver disease | 0.74 | 1 | 0.389 |
| Full cohort | Septic shock | 5.58 | 1 | 0.018 |
| Full cohort | nephrotoxic drugs | 0.08 | 1 | 0.774 |
| Full cohort | baseline creatinine | <0.01 | 1 | 0.979 |
| Full cohort | MAP | 5.75 | 1 | 0.017 |
| Full cohort | APACHE II | 20.40 | 1 | <0.001 |
| Full cohort | GLOBAL | 70.62 | 16 | <0.001 |

| **Model** | **Variable** | **χ²** | **df** | **p-value** |
| --- | --- | --- | --- | --- |
| Landmark (day 7) | group | 10.50 | 3 | 0.015 |
| Landmark (day 7) | gender | 0.18 | 1 | 0.675 |
| Landmark (day 7) | age | 1.74 | 1 | 0.187 |
| Landmark (day 7) | BMI | 2.67 | 1 | 0.102 |
| Landmark (day 7) | COPD/asthma | 0.90 | 1 | 0.343 |
| Landmark (day 7) | hypertension | 0.74 | 1 | 0.389 |
| Landmark (day 7) | diabetes | 1.53 | 1 | 0.216 |
| Landmark (day 7) | cancer | 0.36 | 1 | 0.546 |
| Landmark (day 7) | chronic liver disease | 2.84 | 1 | 0.092 |
| Landmark (day 7) | Septic shock | 0.47 | 1 | 0.495 |
| Landmark (day 7) | nephrotoxic drugs | 0.03 | 1 | 0.861 |
| Landmark (day 7) | baseline creatinine | 0.87 | 1 | 0.352 |
| andmark (day 7) | MAP | 1.42 | 1 | 0.233 |
| Landmark (day 7) | APACHE II | 3.77 | 1 | 0.052 |
| Landmark (day 7) | GLOBAL | 27.70 | 16 | 0.034 |

The full‑cohort model showed severe violation (p < 0.001); the landmark model also violated the assumption (p = 0.034)

Abbreviations: APACHE II, acute physiologic and chronic health evaluation II; SOFA, sequential organ failure assessment
